# ICOS-ICOSL pathway enhances NKT-like cell antiviral function in pregnant women with COVID-19

**DOI:** 10.7150/ijms.95952

**Published:** 2024-07-16

**Authors:** Lu Zong, Yuanling Zheng, Xiaojing Yu, Xiaoran Dai, Ruoyu Huang, Guoxiu Yan, Yuanhong Xu, Meijuan Zheng

**Affiliations:** 1 Department of Clinical Laboratory, First Affiliated Hospital of Anhui Medical University, Hefei, Anhui, China.; 2 Anhui Medical University, Hefei, Anhui, China.; 3 Department of Clinical Laboratory, Anhui Provincial Maternity and Child Health Hospital, Hefei, China.

**Keywords:** COVID-19, CD3^+^CD56^+^ NKT-like cells, Pregnancy, ICOS

## Abstract

**Objective:** The immune response initiated by SARS-CoV-2 infection in pregnancy is poorly elucidated. We aimed to access and compare the antiviral cellular responses and lymphocytes activation between healthy pregnancies and pregnant women infected with SARS-CoV-2.

**Methods:** We detected the immunological changes of lymphocytes in peripheral blood of healthy non-pregnant women, non-pregnant women with COVID-19, healthy pregnant women, pregnant women with COVID-19 and convalescent group by flow cytometry. *In vitro* blockade was used to identify NKT-like cell activation through ICOS-ICOSL pathway.

**Results:** We found that CD3^+^CD56^+^ NKT-like cells decreased significantly in COVID-19 positive pregnant women compared to healthy pregnant women. NKT-like cells of pregnant women expressed higher level of activating receptors CD69 and NKp46 after SARS-CoV-2 infection. Particularly, they also increased the expression of the co-stimulatory molecule ICOS. NKT-like cells of pregnant women with COVID-19 up-regulated the expression of IFN-γ, CD107a and Ki67. Meanwhile, we found that ICOSL expression was significantly increased on pDCs in pregnant women with COVID-19. Blocking ICOS *in vitro* significantly decreased the antiviral activity of NKT-like cells in COVID-19 positive pregnant women, suggesting that ICOS-ICOSL may play an important role in the virus clearance by NKT-like cells.

**Conclusions:** During SARS-CoV-2 infection, NKT-like cells of pregnant women activated through ICOS-ICOSL pathway and played an important role in the antiviral response.

## Introduction

As a highly pathogenic virus, SARS-CoV-2 infection can lead to acute immune activation and long-term clinical sequelae 1. During infection, SARS-CoV-2 can cause changes in the autophagolysosomal pathway, in which ORF7a can evade the degradation of host autophagic lysosomes 2, 3. While pregnancy develops a special immunological challenge because a genetically nonself fetus must be supported within the female 4. Immune tolerance at the maternal-fetal interface during pregnancy is important for maintaining systemic immune homeostasis 5. The immune response of pregnant women to SARS-CoV-2 infection remains to be explained. Studies showed that symptomatic pregnant women had higher IgG, IgM, and IgA titers than the asymptomatic ones 6, 7. For cellular immune responses, the level of NK cell and γδ T cell activation is comparable between SARS-CoV-2 infected pregnant women and healthy pregnancies 8. Previous data show that pregnant women with COVID-19 could induce virus-specific T cell responses and tolerogenic myeloid dendritic cells activation in pregnant women 9. It is urgent to study the antiviral cellular responses and lymphocytes activation between healthy pregnancies and SARS-CoV-2 infected pregnant women.

Human CD3^+^CD56^+^ NKT-like cells are a distinct population of T cells which exhibit both innate and adaptive immunity characters. NKT-like cells participate in antiviral immune responses and show high killing potential against tumor cells 10, 11. In HIV infections, activated NKT-like cells could effectively reduce the viral load in serum by secreting IFN-γ and CD107a 12. Moreover, recovered hepatitis E virus (HEV) patients exhibited more activated phenotype of CD16^+^ NKT-like cells in their peripheral blood, indicating NKT-like cells may also contribute to controlling of HEV infection 13. In COVID-19, previous reports have showed that there was a reduced percentage of CD3^+^CD56^+^ NKT like cells in the peripheral blood of severe group compared to those of mild group, and circulating NKT-like cell frequency could be identified as a predictive biomarker for clinical outcome 14, 15. The phenotypic and functional changes of NKT-like cells in COVID-19 have not been elucidated, nor have they been reported in pregnant women infected with COVID-19.

Inducible costimulatory (ICOS), an important member of CD28 family, is usually expressed on activated T cells. ICOS ligand (ICOSL), which belongs to B7 family, is expressed on B cells, professional antigen-presenting cells and nonlymphoid cells. ICOS signaling is particularly important for complete germinal center development, T cell-dependent B cell responses and stimulating effector T cell responses. ICOS signaling inactivates the transcription factor FOXO1 to promote Tfh cell differentiation 16. ICOS pathway in CD8^+^ T cells contribute to the formation of tissue-resident memory T cells 17. Besides, ICOS pathway helps to improve the persistence of CD8^+^ T cells in CAR-T therapy of tumor 18. In our study, we found ICOS of NKT-like cells engaged with ICOSL on plasmacytoid DCs, and promote the antiviral function of NKT-like cells in pregnant women with COVID-19.

## Methods

### Sample collection and treatment

Blood samples of pregnant women and non-pregnant women with COVID-19 were collected from the First Affiliated Hospital of Anhui Medical University from December 2022 to May 2024. Non-pregnant women with COVID-19 (COVID-19 group) and pregnant women with COVID-19 (PCOVID-19 group) were diagnosed according to SARS-CoV-2 RNA positive by reverse transcriptase polymerase chain reaction (Daan, China). All enrolled COVID-19 positive women were symptomatic. The main symptoms were high fever (>37.5°C), respiratory infections and muscle soreness. Blood samples from COVID-19 group and PCOVID-19 group were collected 2-3 days after the onset of the disease. Convalescent pregnant women (PConvalescent group) were chosen at least 2 months after the SARS-CoV-2 RNA test turned negative. PConvalescent group didn't have infection symptoms nor fever. The healthy pregnant women (PHC group) samples were collected from women who underwent antenatal examination and tested negative for SARS-CoV-2 and other infectious diseases in the First Affiliated Hospital of Anhui Medical University and Anhui Provincial Maternity and Child Health Hospital. Healthy non-pregnant women (HC group) and COVID-19 group were all women of reproductive age.

After 2ml of peripheral blood was mixed in an EDTA anticoagulant tube, neutrophils, white blood cells, lymphocytes, monocytes, platelets and hemoglobin were detected by XN9000 automatic detector (Sysmex, Japan) and its corresponding reagents (all from Sysmex, Japan). Peripheral blood mononuclear cells were obtained by ficoll density gradient centrifugation or hemolysin treatment. Then the cells were labeled with antibodies for flow cytometry detection and, otherwise, were mixed with frozen solution and preserved at -80°C until the day of the experiment.

### Study approval

The studies involving human participants were reviewed and approved by the Medical Ethics Committee of The First Affiliated Hospital of Anhui Medical University. The patients/participants provided their written informed consent to participate in this study (Quick-PJ 2023-13-39).

### Flow cytometry

For surface marker detection, the obtained PBMCs were labeled with flow cytometric antibodies, washed and detected on flow cytometer (BD FACSCanto plus, BD Biosciences). For intracellular staining, PBMCs were stimulated by PMA (50 ng/ml, Multisciences, China) and ionomycin (1 μg/ml, Sigma, USA) at 37 °C for 3.5 hours. Monensin (2.5 μg/ml; Multisciences, China) was added at the last hour. Then cells were washed, labeled with surface markers, fixed, permeabilized, stained with cytokine antibodies, and finally detected by flow cytometer. The data were analyzed by FlowJo VX analysis. The antibodies used in the study were as follow: Alexa Fluor 488-anti-NKG2A, PerCP-Cy5.5-anti-CD69, BV510-anti-Tim-3, PE-Cy7-anti-CD107a, BV605-anti-TIGIT, FITC-anti-ICOS, PerCP-Cy5.5-anti-HLA-DR, PE-anti-ICOSL and PerCP-Cy5.5-anti-CD3, APC-R700-anti-CD123 were from Biolegend. BV421-anti-PD-1, BV510-anti-CD3, PE-Cy7-anti-CD3, APC-R700-anti-CD56, BV605-anti-CD3, APC-H7-anti-CD8, BV510-anti-Granzyme B, BB700-anti-IFN-γ, BV510-anti-CD4, PE-anti-NKp46, BV605-anti-CD14, PE-CY7-anti-CD16, BV421-anti-CD80, BV421-anti-CD19, PE-anti-Annexin V, PerCP-Cy5.5-anti-7AAD, BV421-anti-Granzyme B, APC-anti-Ki67, PerCP-Cy5.5-anti-IFN-γ, APC-anti-CD107a, BV421-anti-CD56, APC-R700-anti-CD8, BV421-anti-CD275, PE-anti-CD123, APC-anti-CD56, APC-anti-CD20, APC-anti-CD19, APC-anti-CD3 and APC-anti-CD66b were from BD Biosciences. FITC-anti-NKG2A was from Miltenyi Biotec. FITC-anti-CD11c was from eBioscience.

### Cytometric bead array (CBA) assay

The soluble cytokines were detected with the detection kit of IFN-γ/ IL-1β/ IL-2/ IL-4/ IL-5/ IL-6/ IL-8/ IL-10/ IL-12p70/ IL-17A/ IL-17F/ IL-22/ TNF-α/ TNF-β (QuantoBio, China). The samples are processed in accordance with the instructions and then detected on BEAMDIAG (China).

### *In vitro* blocking assay

First, mouse anti-human CD3(5μg/ml, Clone: OKT3, Biolegend) was added in 96-well plates and incubated overnight at 4°C. Then the liquid was discarded on the second day. The isolated PBMCs were suspended in 1640 culture medium (1.5% HEPES, 1% streptomycin/ penicillin, 10% fetal bovine serum). Anti-ICOS (2.5μg/ml, Clone: 669214, biotechne) or mouse IgG was added in the corresponding wells. The cells were cultured at 37°C, 5% CO_2_ for 72 hours. Then the cells were harvested and washed by PBS. Later, the cells underwent conventional cytokine antibodies labeling, and were finally tested by flow cytometer. Considering the internalization of TCR/CD3 during T cell activation with anti-CD3, we chose to label the fluorescent antibody (PerCP-Cy5.5-anti-CD3, Clone: HIT3a, Biolegend) differently from the stimulated anti-CD3 clone.

### Data analysis

All data were represented as mean ± standard error of the mean (SEM) and analyzed by GraphPad Prism 9.4.1 software. According to the distribution of data, it was determined that one-way ANOVA or the Kruskal-Walli's test was used for data analysis, and paired *t*-tests were used in the blocking assay. *P* value< 0.05 was considered statistically significant.

## Results

### Pregnant women with COVID-19 did not get cytokine storms

A total of 18 healthy non-pregnant women, 13 non-pregnant women with COVID-19, 37 healthy pregnant women, 32 pregnant women with COVID-19 and 30 convalescent pregnant women were enrolled in this study. The clinical characteristics of healthy non-pregnant women (HC group), non-pregnant women with COVID-19 (COVID-19 group), healthy pregnant women (PHC group), pregnant women with COVID-19 (PCOVID-19 group), and convalescent pregnancies (PConvalescent group) are shown in Table 1. The minimum gestational age of the selected samples was 6w^+4^ and the maximum gestational age was 38w^+5^. Pregnant women in the second and third trimester were the majority of the samples selected in the experiment. The level of alanine aminotransferase and aspartate aminotransferase didn't increase in pregnant women with COVID-19, suggesting the liver function was not impaired. At the same time, we found that the D-dimer of pregnant women was significantly higher than that of healthy women, but there was no difference between infected pregnant women and healthy pregnant women. The immune status of pregnant women changes significantly. A pro-inflammatory state predominates during the first trimester mainly to protect the mother, followed by the second trimester which is predominated by the anti-inflammatory state, and lastly, the inflammatory environment returns to induce childbirth in the third trimesters 19-21. Although the anti-inflammatory state can promote the development of the fetus, it also leaves the mother in an immunosuppressive state and more susceptible to infection 22, 23. Most of the previous literature has reported the cytokine storm in COVID-19 patients, and it is more obvious in severe patients 24, 25. We detected the level of serum cytokines during the three trimesters and found that the cytokine storms did not occur in pregnant women with COVID-19 (**Figure S1A-N**). Except for IL-8 decreased in the second trimester after infected with SARS-CoV-2, IL-2, TNF-α, TNF-β, IFN-γ, IL-4, IL-5, IL-6, IL-10, IL-17A, IL-17F, IL-22, IL-1β and IL-12p70 didn't change in the three trimesters between healthy pregnancies and pregnant women with COVID-19.

### CD3^+^CD56^+^ NKT-like cells decrease dramatically in pregnant women with COVID-19 compared with healthy pregnant women

**Figure 1:**
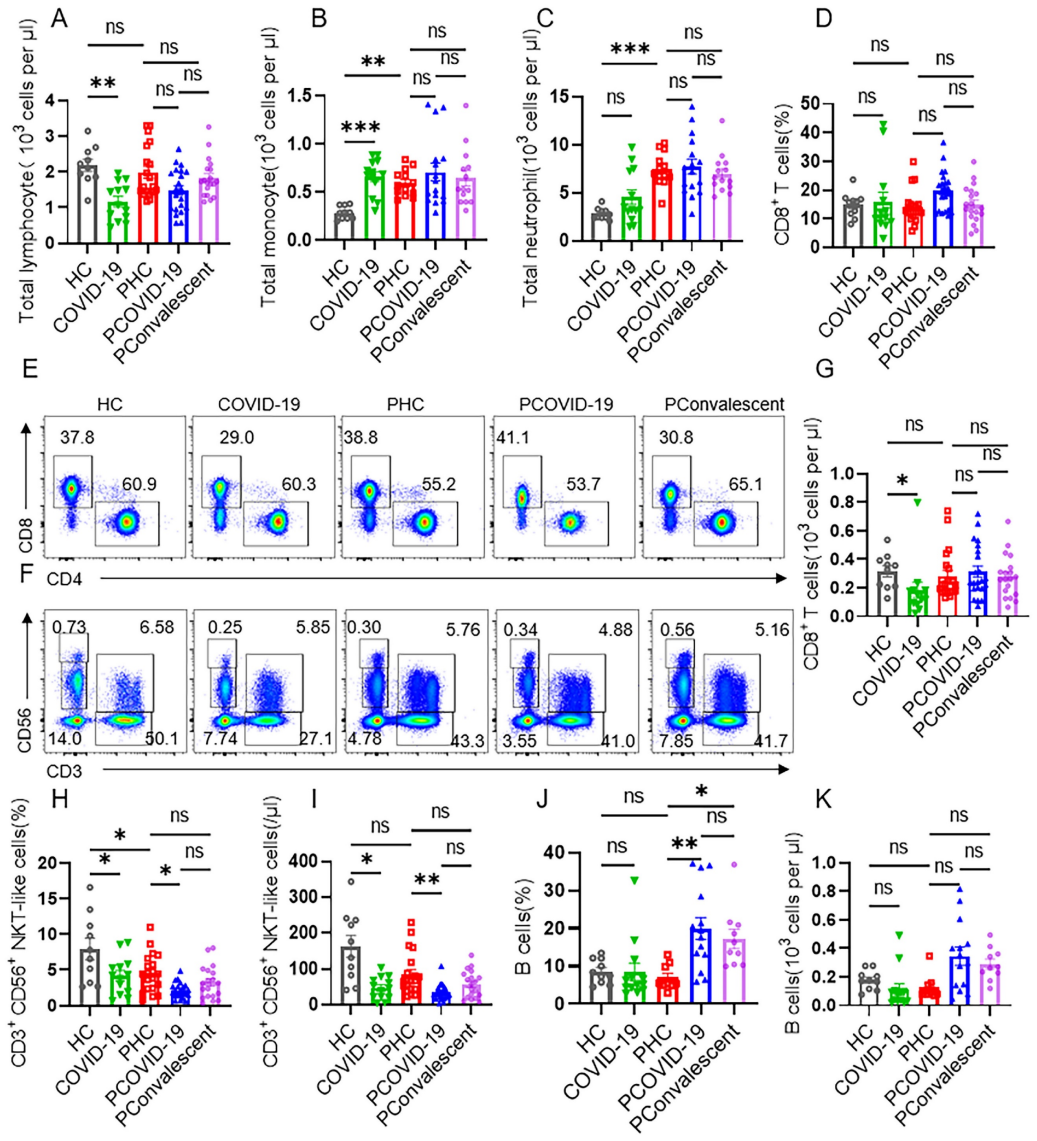
** CD3^+^CD56^+^ NKT-like cells decrease dramatically in pregnant women with COVID-19 compared with healthy pregnant women.** (**A-C**) The cellularity of lymphocytes, monocytes and neutrophils in peripheral blood of healthy control (HC group), non-pregnant women with COVID-19 (COVID-19 group), healthy pregnant women control (PHC group), pregnant women with COVID-19 (PCOVID-19 group) and convalescent patients (PConvalescent group). (**E-F**) Representative flow cytometry graphs of CD4^+^ T cells, CD8^+^ T cells, CD56^bri^ NK cells, CD56^dim^ NK cells, CD3^+^CD56^+^ NKT-like cells and T cells. (**D, G-K**) Flow cytometry detected the percentages and absolute number of (**D and G**) CD8^+^ T cells, (**H-I**) CD3^+^CD56^+^ NKT-like T cells and (**J-K**) CD19^+^ B cells among HC group, COVID-19 group, PHC group, PCOVID-19 group, and PConvalescent group. Data are shown as mean ± SEM. One-way ANOVA was used to determine statistical significance. *P< 0.05; **P <0.01; ***P <0.001; ns, not significant.

Despite the fact that lymphocytes decreased seriously in non-pregnant people during COVID-19, we found there was no statistical difference in the cellularity of total lymphocytes among PHC group, PCOVID-19 group and PConvalescent group (**Figure 1A**). After pregnancy, there is a physiological increase in monocytes and neutrophils (**Figure 1B-C**), which is consistent with other reports 26. Compared to PHC group, the numbers of monocytes and neutrophils didn't change in pregnant women after SARS-CoV-2 infection (**Figure 1B-C**). T cells are important cells against virus infection in adaptive immunity. We found no changes in the proportion and cellularity of T cells, CD4^+^ T cells and CD8^+^ T cells between PHC group and HC group. The proportion and absolute count of total T cells and CD4^+^ T cells in COVID-19 group showed a significant decrease compared to HC group. But there were not statistically significant among the three pregnant groups (**Figure S2A-D**). Despite an increased absolute count of CD8^+^ T cells was observed, but the proportion count of CD8^+^ T cells did not differ significantly in the non-pregnant groups, possibly because of the downward trend in the total lymphocytes number (**Figure 1D-E and 1G**). COVID-19 is an acute disease of rapid onset. Therefore, we detected the cells of innate immunity next. We found that the proportion and cellularity of the CD56^bri^ natural killer (NK) cells and CD56^dim^ NK cells were comparable among the five groups (**Figure S2E-H**). However, the proportion and absolute count of NKT-like cells of the COVID-19 group were significantly lower than those of HC group (**Figure 1F and 1H-I**). The proportion of NKT-like cells in PHC group also showed a slight decrease compared to HC group, but there was no difference in absolute counts. Combined with these two effects, the proportion and absolute count of NKT-like cells in infected COVID-19 positive pregnant women showed a more obvious downward trend compared with the PHC group (**Figure 1F and 1H-I**). In addition, we found that the number and proportion of B cells in PCOVID-19 group were higher than those in PHC group, indicating an activated humoral immune response after SARS-CoV-2 infection (**Figure 1J-K**).

### NKT-like cells in pregnant women with COVID-19 express high level of co-stimulating receptor ICOS

**Figure 2:**
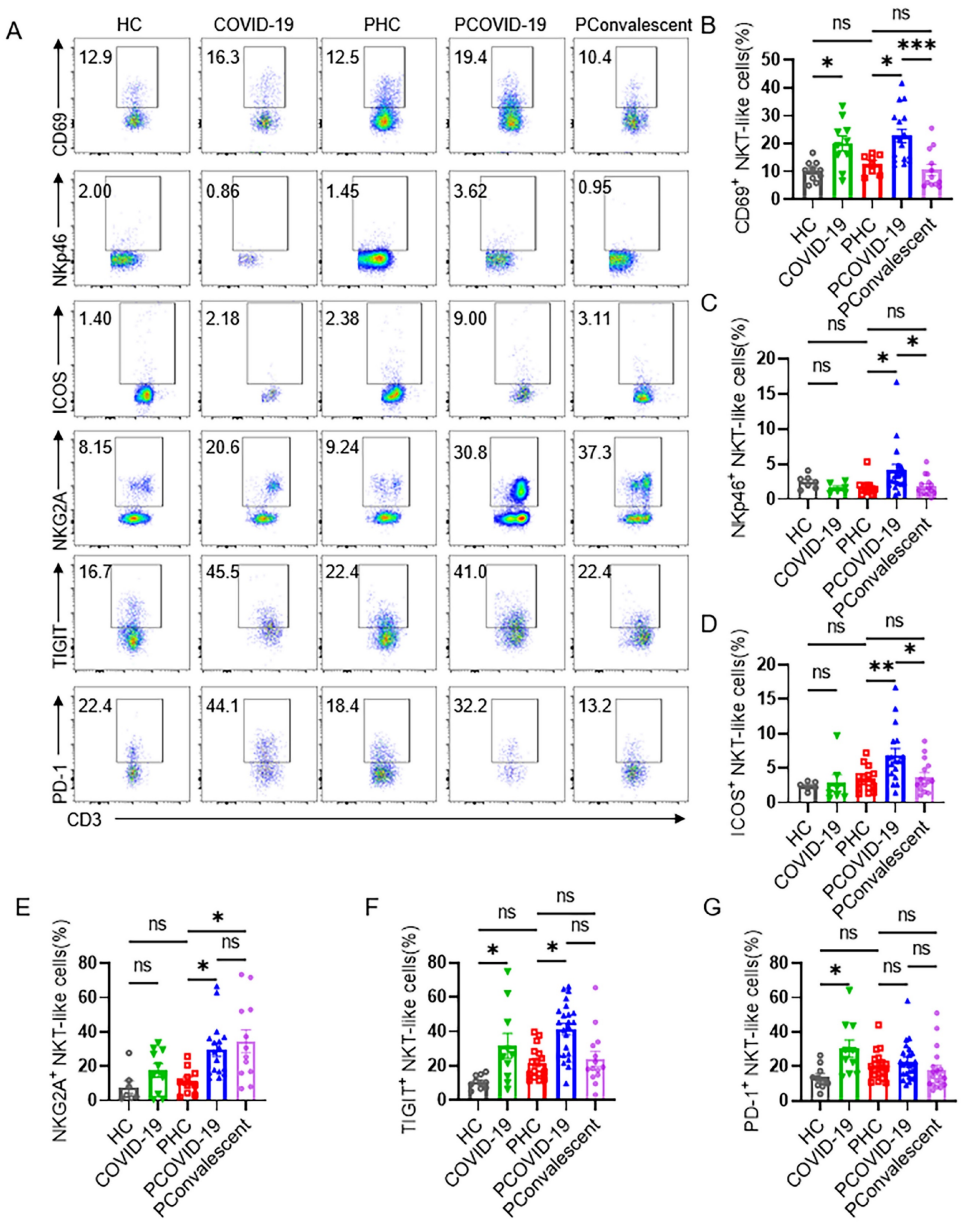
** The phenotype of CD3^+^CD56^+^ NKT-like cells in COVID-19 positive pregnant women.** (**A**) The representative flow cytometry graphs of CD69^+^, NKp46^+^, ICOS^+^, NKG2A^+^, TIGIT^+^, PD-1^+^ NKT-like cells among the HC group, COVID-19 group, PHC group, PCOVID-19 group and PConvalescent group. (**B-G**) The expression of (**B**) CD69, (**C**) NKp46, (**D**) ICOS, (**E**) NKG2A, (**F**) TIGIT and (**G**) PD-1 on NKT-like cells among the HC group, COVID-19 group, PHC group, PCOVID-19 group and PConvalescent group. Results are shown as mean ± SEM. One-way ANOVA was used to determine statistical significance. *P< 0.05; **P <0.01; ***P <0.001; ns, not significant.

Further, we detected the phenotype of CD3^+^CD56^+^ NKT-like cells and found that the expression of activating receptor CD69, increased remarkably on NKT-like cells of pregnant women with COVID-19 compared with PHC group, non-pregnant women infected was also significantly higher than that in HC group (**Figure 2A-B**). NKp46, one of the important NK cell receptors, was also expressed higher on NKT-like cells in PCOVID-19 group, showing an activated state (**Figure 2A and 2C**). Co-stimulatory molecule ICOS augments T cell differentiation and cytokine secretion, and provides critical signals for antibody production 27, so we evaluated ICOS and found NKT-like cells of pregnant women increased the expression of the ICOS during COVID-19 (**Figure 2A and 2D**). In addition, we also found that there was no difference in the expression of ICOS on CD56^bri^ NK, CD56^dim^ NK cells, CD4^+^ T and CD8^+^ T cells between PCOVID-19 group and PHC group (**Figure S3A-D**). Lymphocytes increased their co-inhibitory receptor expression to avoid excessive activation and subsequent apoptosis in COVID-19 and other viral infections 28-30. In our study, we also observed that the expression of inhibitory receptors NKG2A and TIGIT on NKT-like cells increased significantly in pregnant women with COVID-19 compared with PHC group (**Figure 2A, 2E-F**). There was no significant difference in the expression of PD-1 among the three pregnant groups (**Figure 2G**). Moreover, we found that the immunophenotype of PHC group was comparable with that of HC group, indicating the physiology of pregnancy did not significantly change the immune status of NKT-like cells.

### Escalating antiviral function and proliferation ability of NKT-like cells in pregnant women with COVID-19

**Figure 3:**
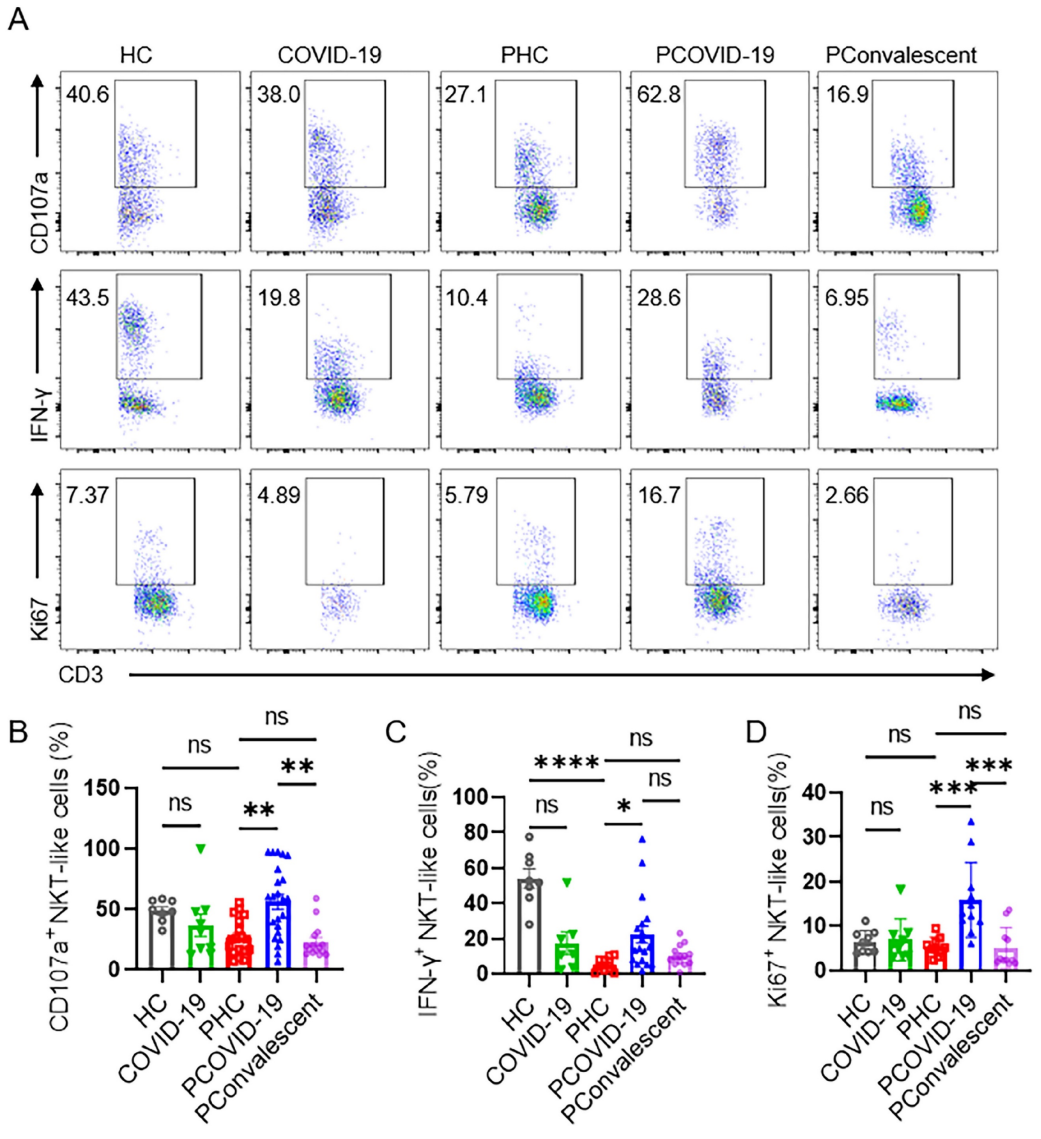
** NKT-like cells of pregnant women enhanced their antiviral ability and proliferation rate after SARS-CoV-2 infection.** (**A**) The representative flow cytometry graphs of CD107a^+^ NKT-like cells, IFN-γ^+^ NKT-like cells and Ki67^+^ NKT-like cells among the HC group, COVID-19 group, PHC group, PCOVID-19 group, PConvalescent group. (**B-D**) The expression of (**B**) CD107a, (**C**) IFN-γ and (**D**) Ki67 on NKT-like cells among the HC group, COVID-19 group, PHC group, PCOVID-19 group, PConvalescent group. Results are shown as mean ± SEM. One-way ANOVA was used to determine statistical significance. *P< 0.05; **P <0.01; ***P <0.001; ****P <0.001; ns, not significant.

**Figure 4:**
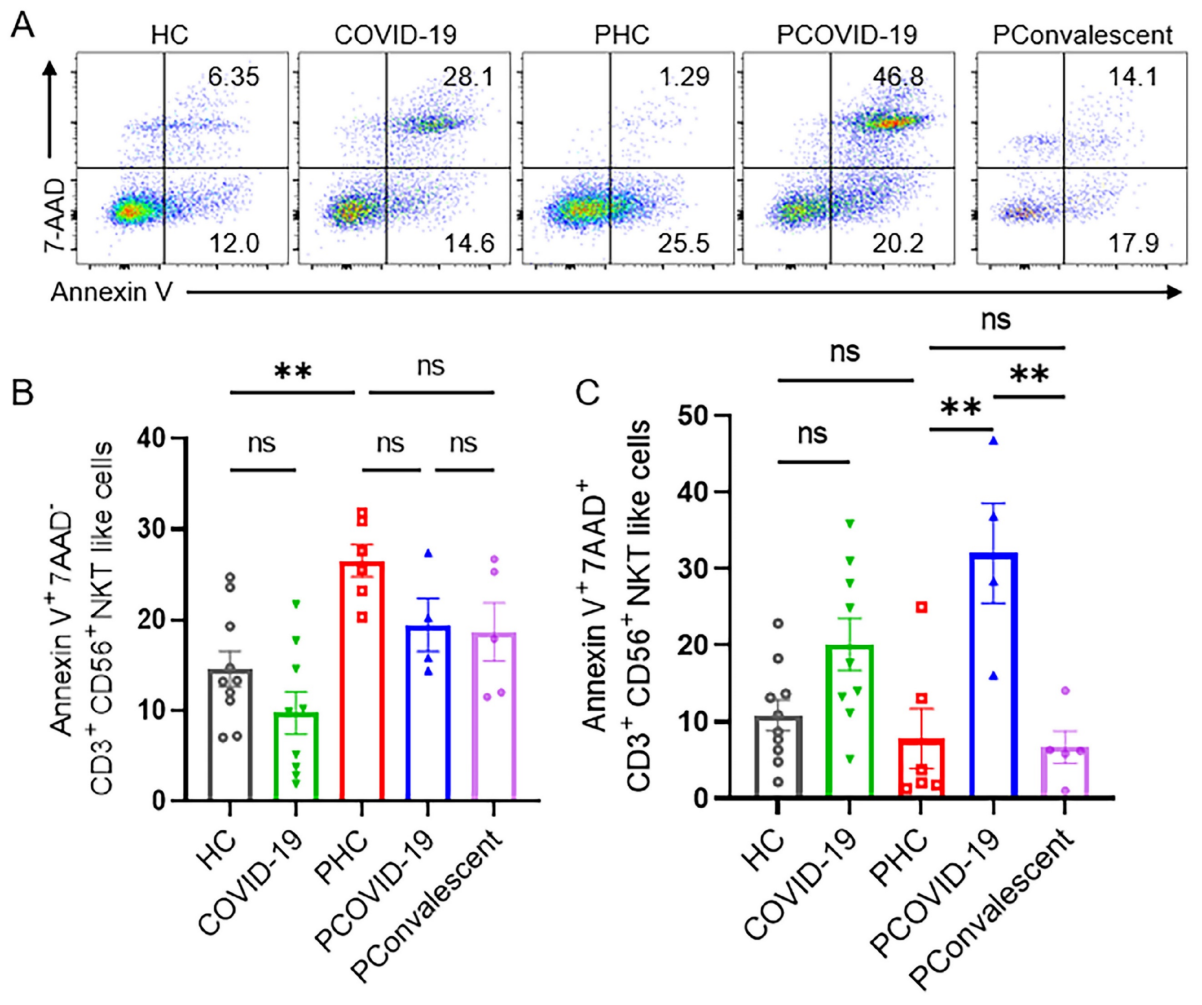
** The apoptotic levels of NKT-like cells among the HC group, COVID-19 group, PHC group, PCOVID-19 group and PConvalescent group.** (**A**) Representative flow graphs of Annexin V and 7-AAD staining of NKT-like cells. (**B-C**) The statistical diagram of (**B**) Annexin V^+^7-AAD^-^ NKT-like cells and (**C**) Annexin V^+^7-AAD^+^ NKT-like cells among the HC group, COVID-19 group, PHC group, PCOVID-19 group and PConvalescent group. Results are shown as mean ± SEM. One-way ANOVA was used to determine statistical significance **P <0.01; ns, not significant.

We monitored the degranulation ability and IFN-γ secretion of NKT-like cells in pregnant patients during SARS-CoV-2 infection. As an important marker of cytolysis, we found that CD107a expressed on NKT-like cells of PCOVID-19 group significantly increased compared with healthy pregnant women, and recovered in convalescence (**Figure 3A-B**). IFN-γ expressed by NKT-like cells was significantly decreased in women after pregnancy (**Figure 3A and 3C**). Besides, compared with the PHC group, IFN-γ secreted by NKT-like cells also showed an increased trend in PCOVID-19 group, and recovered in convalescence (**Figure 3A and 3C**). In addition, proliferation level is an important marker for lymphocytes activation, so we measured the proliferation marker Ki67. We found NKT-like cells of pregnant women showed high proliferation ability after SARS-CoV-2 infection and recovered in convalescent stage (**Figure 3A and 3D**). At the same time, we detected changes of apoptosis levels in COVID-19 positive pregnant women. Annexin V and 7-AAD staining showed NKT-like cells of pregnant women with COVID-19 got an early apoptotic level comparable with PHC group. However, we also found that the level of early apoptosis in healthy pregnant women was significantly higher than that in healthy non-pregnant women (**Figure 4A-B**). Moreover, we observed a significant elevation on late apoptosis levels in PCOVID-19 group compared to PHC group, which subsequently returned to normal levels in the convalescence (**Figure 4A and 4C**).

CD56^bri^ NK cells are abundant cytokine producers after activation. However, we didn't observe significant differences in the expression of CD107a and Ki67 of this subpopulation among the five groups (**Figure S4A, S4C**). The expression of IFN-γ by CD56^bri^ NK cells decreased after pregnancy in a physiological manner (**Figure S4B**), the same as CD56^dim^ NK cells, CD4^+^ T and CD8^+^ T cells (**Figure S4E, S5B, S5E**). CD56^dim^ NK cells, the main subgroup of NK cells, have robust cytotoxicity 31. We found that the level of CD107a were higher on CD56^dim^ NK cells of COVID-19 positive pregnant women than those of PHC group, and decreased in convalescent group (**Figure S4D**). CD4^+^ T and CD8^+^ T cells showed the similar trend in the expression of CD107a as CD56^dim^ NK cells (**Figure S5A, S5D**). Ki67 expressed by CD56^dim^ NK cells, CD4^+^ T and CD8^+^ T cells didn't change after COVID-19 (**Figure S4F, S5C, S5F**). Above data demonstrated that NKT-like cells in pregnant women escalate their antiviral function and proliferation ability, and may play an important role in defending against SARS-CoV-2 infection.

### ICOSL on plasmacytoid DCs engaged with NKT-like cells in COVID-19 positive pregnant women

**Figure 5:**
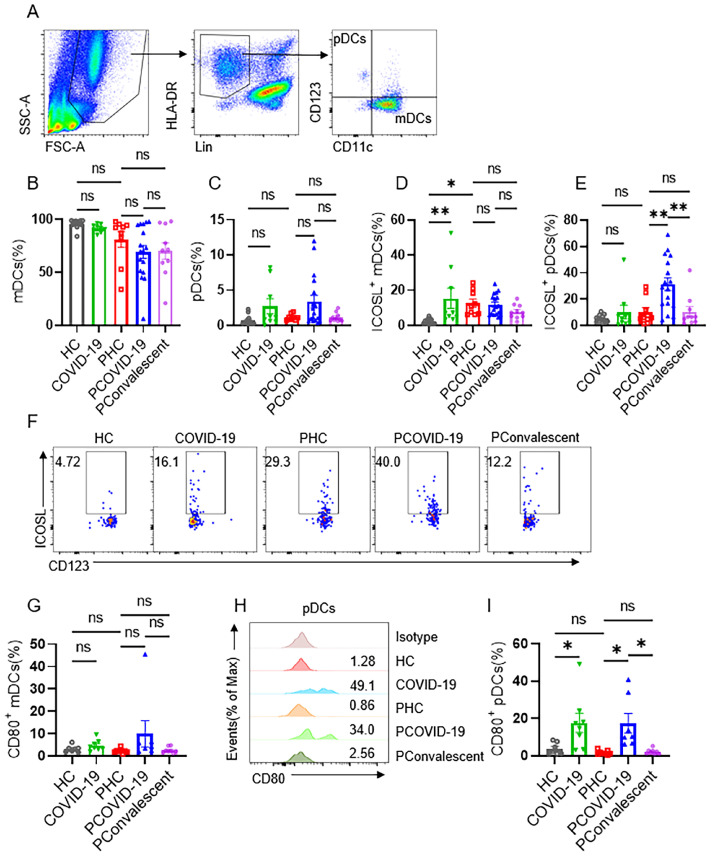
** Expression of ICOSL on plasmacytoid DCs and myeloid DCs in COVID-19 positive pregnant women.** (**A**) Gating strategy for myeloid DCs (mDCs): Lin^-^ (CD3, CD19, CD20, CD56, CD66b) HLA-DR^+^CD123^-^CD11c^+^ and plasmacytoid DCs (pDCs): Lin^-^ HLA-DR^+^CD123^+^CD11c^-^. (**B-C**) The proportion of (**B**) mDCs and (**C**) pDCs among the HC group, COVID-19 group, PHC group, PCOVID-19 group, PConvalescent group. (**D-E**) The expression of ICOSL on (**D**) mDCs and (**E**) pDCs among the HC group, COVID-19 group, PHC group, PCOVID-19 group, PConvalescent group. (**F**)The representative flow cytometry graphs of ICOSL on pDCs among the HC group, COVID-19 group, PHC group, PCOVID-19 group and PConvalescent group. Expression of CD80 on (**G**) mDCs and (**I**) pDCs among the HC group, COVID-19 group, PHC group, PCOVID-19 group and PConvalescent group. (**H**) Representative histogram graphs for the expression of CD80 on pDCs. Results are shown as mean ± SEM. One-way ANOVA was used to determine statistical significance. *P <0.05; **P <0.01; ns, not significant.

ICOS-ICOSL pathway is important for stimulating effector immune response. We further examined the expression of ICOSL in COVID-19 positive pregnant women. We first detected monocytes and found the proportion of CD14^+^CD16^-^ (classical), CD14^+^CD16^+^ (intermediate) and CD14^-^CD16^+^ (non-classical) monocytes group didn't change among the five groups (**Figure S6A-D**). Nor did the expression of ICOSL on monocytes (**Figure S6E-G**). Then, we measured dendritic cells (DCs), which contains two main subsets: myeloid DCs (mDCs) and plasmacytoid DCs (pDCs). We found that the ratio of mDCs and pDCs did not differ among the five groups (**Figure 5A-C**). The expression of ICOSL in mDCs in COVID-19 group was significantly higher than that in HC group, and ICOSL expression in mDCs in PHC group was significantly higher than that in HC group (**Figure 5D**). But there was no difference in the expression of ICOSL on mDCs among the three pregnant groups (**Figure 5D**). While ICOSL expression on pDCs of PCOVID-19 group increased significantly compared with PHC group, and returned to normal level in convalescent group (**Figure 5E-F**). At the same time, we also detected the expression of CD80 on DCs, and found that CD80 showed a significant increase on pDCs of pregnant and non-pregnant patients infected with COVID-19 (**Figure 5H-I**). This indicates that pDCs activate during COVID-19 invasion, but this activation is not specific to pregnant women. These data suggest that pDCs may activate NKT-like cells through ICOS-ICOSL pathway in pregnant women with COVID-19.

### ICOS blockade reduce the effector function of NKT-like cells in COVID-19 positive pregnant women

**Figure 6:**
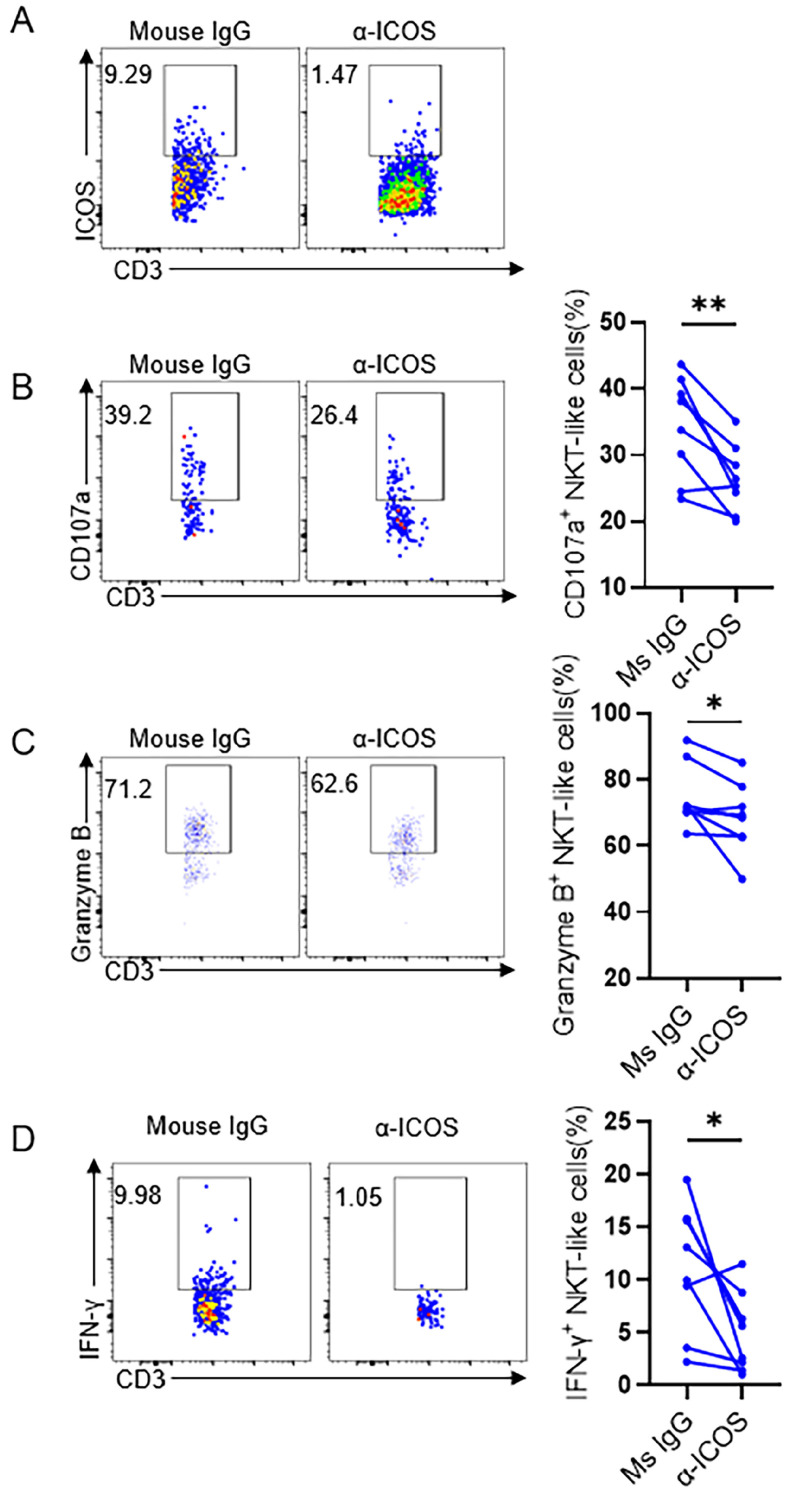
** ICOS blockade reduced the cytokine secretion level of NKT-like cells in COVID-19 positive pregnant women.** (**A**) *In vitro* blocking effect of anti-ICOS on NKT-like cells. (**B-D**) The frequency of (**B**) CD107a^+^ NKT-like cells, (**C**) Granzyme B^+^ NKT-like cells and (**D**) IFN-γ^+^ NKT-like cells of COVID-19 positive pregnant women in the presence of anti-ICOS mAb or isotype mAb. Data are shown as mean ± SEM. Paired Student's *t*-tests were performed. *P< 0.05; **P <0.01; ns, not significant.

The up-regulation of ICOS on NKT-like cells and the increased expression of the ligand ICOSL on pDCs suggest that ICOS-ICOSL pathway may be a potential way of NKT-like cell activation. To assess this hypothesis, ICOS blockade were taken on isolated PBMCs which were then stimulated by anti-CD3 *in vitro* for 72 hours in pregnant women with COVID-19 (**Figure 6A**). We found that the expression of CD107a, granzyme B and IFN-γ on NKT-like cells was reduced in the presence of anti-ICOS blocking antibodies (**Figure 6B-D**), suggesting that ICOS-ICOSL pathway indeed play an important role in stimulating effector response of NKT-like cells for pregnant women defending against SARS-CoV-2 infection.

## Discussion

We found NKT-like cells of pregnant women exhibited a more activated phenotype and stronger cytotoxic ability after SARS-CoV-2 infection. ICOS is well known for its involvement in B cell differentiation and providing critical T cell help to B cells. We found NKT-like cells may engage with pDCs through ICOS-ICOSL pathway in pregnant women with COVID-19. Our study provides insights in clinical treatment and vaccine design strategies for pregnant women.

Previous reports show that the body develops severe cytokine storms after SARS-CoV-2 infection and manifests multiple organ damage in fatal cases 24, 32. While pregnant women, show a more complicated humoral immune response during COVID-19. As the time progressed from the first trimester to the second trimester of pregnancy, Th1 cell-mediated immunity changes to Th2 cell dominated environment. Both pro-inflammatory and anti-inflammatory immunity were activated in parallel during SARS-CoV-2 infection, thus avoiding a potential pro-inflammatory cytokine storm. This coincidence of immunomodulation during pregnancy may protect pregnant women with COVID-19 from cytokine storms and the development of acute respiratory distress syndrome 33. We didn't find any Th1/Th2/Th17 cytokine level changes in our study. But Valeria Garcia-Flores *et al.* showed that SARS-CoV-2-positive pregnant women had a mild inflammatory response, possibly due to the presence of severely ill patients 34.

B cells play an important role in the defense against viral infections such as SARS-CoV-2 35. As disease severity progress, pregnant women infected with COVID-19 got an increase in naïve, immature B-cells and a decrease in memory B-cells, whose maturation capacity to form plasma blast cells remains fine 36. For regulatory B cells, on one hand, the frequency of CD24^hi^CD38^hi^ regulatory B cells is reduced in critically ill COVID-19 patients and their ability to secrete IL-10 was also impaired 37. On the other hand, Breg is important for the maintenance of immune tolerance in pregnant women during early pregnancy 38. The combination of these two aspects may develop a compromised anti-SARS-CoV-2 immunity in SARS-CoV-2-positive pregnant women. The function of Breg cells in COVID-19 during pregnancy need to be further explored.

As a bridge between innate and adaptive immunity, NKT-like cells express a high-density TCR-CD3 complex and a low level of co-stimulating molecule CD28, and could mediate cytotoxicity restricted in a non-MHC dependent pathway 39, 40. There is also viewpoint suggesting CD28 appears to play a predominant role in priming T cells during Th2 immune response, whereas ICOS is more likely to regulate effector T cell responses 41. In our study, ICOS-ICOSL pathway can activate NKT-like cells in antiviral response. Another co-stimulatory molecule, CD137, can also induce the expansion and activation of NKT-like cells 42. In the design of CAR-T co-stimulatory domains, the incorporation of ICOS significantly increased the persistence of T cells than the presence of CD28 or CD137 alone 18, 43, 44. The role of other costimulatory signals for NKT cells to exert effector response needs to be further explored.

We found that the cellularity and absolute count of NKT-like cells of COVID-19 positive pregnant women were lowest among the five groups. Although the proliferation of NKT-like cells was increased, pregnancy is physiologically associated with increased levels of early apoptosis, and SARS-CoV-2 infection increased the late apoptosis of NKT-like cells (Annexin V^+^7AAD^+^). The combination of high levels of late apoptosis and physiological changes during pregnancy may ultimately lead to the reduction of NKT-like cells in the peripheral blood of COVID-19 positive pregnant women. Besides, in PConvalescent group, we found that the phenotype and antiviral ability of NKT-like cells did not recover to the level of healthy pregnant women. It has been reported that T cells still showed a slightly enhanced activation and proliferation rate after recovering from COVID-19 for months, indicating that these individuals were in a phase of ongoing restoration of immune homeostasis 45, so the function of NKT-like cells requires long-term dynamic monitoring to find when they return to normal.

Because the large blood volume required to obtain pure pDCs and NKT-like cells by flow sorting is limited by ethics, we cannot obtain sufficient blood volume from pregnant women who have babies. Previous studies have shown that compared with mDCs, pDCs preferentially up-regulate the expression of ICOSL upon virus antigen stimulation 46. Our data also showed ICOSL expression on pDCs increased dramatically in PCOVID-19 group. For mDCs, we found that the expression of ICOSL on mDCs was significantly higher in PHC group compared to HC group, indicating the process of pregnancy can lead to the increased expression of ICOSL on mDCs. While the ICOSL expression between PCOVID-19 group and PHC group showed no difference. We also found that the expression of ICOSL also showed an increased trend in monocytes during COVID-19, although there was no statistical difference. It has been previously reported that circulating monocytes can migrate to tissues and differentiate into dendritic cells and macrophages 20, 47. So we speculate that monocytes may also play an indispensable role in activating lymphocytes through ICOS-ICOSL pathway.

Plasmacytoid dendritic cells are a unique group of cells in the immune system that produce large amounts of type I interferon α to resist viruses 48, 49. pDCs are the dominant IFN-α-producing cells in response to SARS-CoV-2 and can lead to macrophage activation severe COVID-19 patients 50, 51. Studies have also shown that IFN-α is significantly elevated in COVID-19 positive mothers and in cord blood of their neonates 36. It has also been reported that IFN-α released by pDCs can enhance the cytolytic activity of CD56^+^ T cells 52. Therefore, we speculate that pDCs may also enhance the antiviral activity of NKT-like cells by secreting IFN-α in SARS-CoV-2-infected pregnant women, which needs to be further studied.

SARS-CoV-2 infection in pregnant women has also been associated with a mild cytokine response in cord blood 34. Previous studies have shown that in pregnant women infected with COVID-19, IgG levels in peripheral blood and umbilical cord blood are high, and the levels in symptomatic patients are higher than those in asymptomatic patients 6, 8. TNF-α, IFN-α, IFN-γ, and IL-6 were significantly elevated in both the peripheral blood of COVID-19 positive pregnant women and cord blood, which indicate both threat to mother and fetus 36. In addition, unique inflammatory responses against the SARS-CoV-2 are induced at the maternal-fetal interface, and these inflammatory responses are mainly controlled by maternal T cells and fetal stromal cells 34. The immune status changes of cord blood in COVID-19 positive pregnant women are hoped to be further explored in the future.

In conclusion, we found that NKT-like cells activated and enhanced their antiviral activity obviously in SARS-CoV-2 infection during pregnancy. NKT-like cells may play an important role in the resistance to SARS-CoV-2 virus by engaging with pDCs through ICOS-ICOSL pathway. ICOS blockade significantly relieved the antiviral activity of NKT-like cells. Because of the physiological change in pregnant women, pregnant women infected SARS-CoV-2 showed complicated immune statuses. Our study has important clinical guidance for the treatment of COVID-19 in pregnant women.

## Supplementary Material

Supplementary figures.

## Figures and Tables

**Table 1:** Clinical characteristics of healthy non-pregnant women, non-pregnant women with COVID-19, healthy pregnant women, pregnant women with COVID-19 and convalescent group.

	HC n=18	COVID-19 n=13	PHC n=37	PCOVID-19 n=32	PConvalescent n=30	P value
**Age**	34.61 (34.5)	42.92 (41)	30.89 (31)	29.58 (30)	30.53 (30.5)	0.033
**Gestational weeks**						
**First trimester (<13^+6^ w)**			6/37 (16.21%)	7/32 (21.88%)	1/30 (3.33%)	
**Second trimester (14^+0^ w-27^+6^ w)**			18/37 (48.65%)	11/32 (34.37%)	24/30 (80%)	
**Third trimester (28^+0^ w-40^+6^ w)**			13/37 (35.14%)	14/32 (43.75%)	5/30 (16.67%)	
**Comorbidities**						
**Diabetes**	0	0	2/37 (5.41%)	1/32 (3.13%)	1/30 (3.33%)	
**Hypertension**	0	0	1/37 (2.7%)	1/32 (3.13%)	0	
**laboratory index**						
**Alanine aminotransferase, U/L**	16.67 (15)	39.83 (17)	14.17 (10.5)	26.82 (15) ^a^	19.1 (17)	0.0078
**Aspartate aminotransferase, U/L**	23.5 (18.5)	33.58 (31.8)	19.63 (18)	27.35 (20.5)	20.9 (19)	0.1309
**Alkaline phosphatase, U/L**	61.79 (62)	83.72 (76)	98.79 (76)	104.41 (97.5)	64.6 (63.5)	0.0110
**C-reactive protein, mg/L**	2.7 (2.62) ^b^	14.41 (9.9)	4.01 (4.55)	21.59 (6.16)	4.65 (4.51)	0.0012
**D-dimer, mg/L**	0.28 (0.28) ^c^	0.88 (1.03)	1.23 (1)	1.94 (1.57)	1.01 (0.92)	<0.0001

Skewed distribution: Median (IQR). a: Comparison between PCOVID-19 group and PConvalescent group: *P* < 0.05 b: Comparison between HC group and COVID-19 group: *P* < 0.05. c: Comparison between HC group and PHC group: *P* < 0.05

## Abbreviations

HC group: healthy non-pregnant women
COVID-19 group: non-pregnant women with COVID-19
PHC group: healthy pregnant women control
PCOVID-19 group: pregnant women with COVID-19
PConvalescent group: convalescent patients
Inducible costimulatory: ICOS
ICOS ligand: ICOSL
myeloid dendritic cells: mDCs
plasmacytoid dendritic cells: pDCs
