# Supplementary material for: ICOS-ICOSL pathway enhances NKT-like cell antiviral function in pregnant women with COVID-19
Source: Int J Med Sci. 2024 Jul 16;21(10):1890–902. doi: 10.7150/ijms.95952 (PMC11302565; doi:10.7150/ijms.95952)
Supplement: Supplementary file 1 — Supplementary figures. [file ijmsv21p1890s1.pdf]

Supplementary Figures

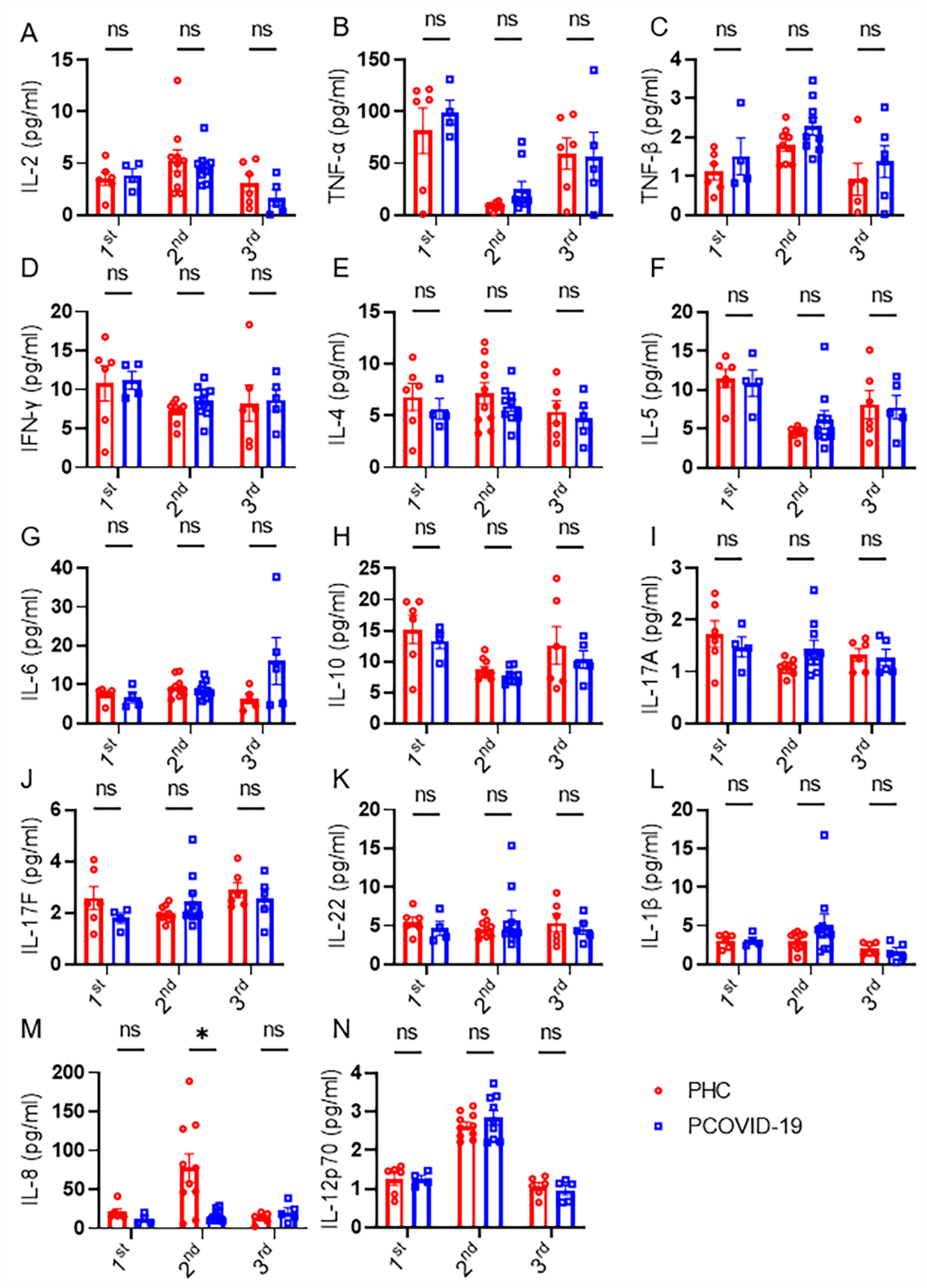

Supplementary Figure 1. Cytokine levels in healthy pregnant women and pregnant women with COVID-19. Comparison of cytokines levels of (A)IL-2, (B)TNF-α, (C)TNF-β, (D)IFN-γ, (E)IL-4, (F)IL-5, (G)IL-6, (H)IL-10, (I)IL-17A, (J)IL-17F, (K)IL-22, (L)IL-1β, (M)IL-8 and (N)IL-12p70 in healthy and COVID-19 positive pregnant women during different periods of pregnancy. Data are shown as mean ± SEM. Multiple unpaired *t*-tests were used to determine statistical significance. *$P < 0.05$; ns, not significant.

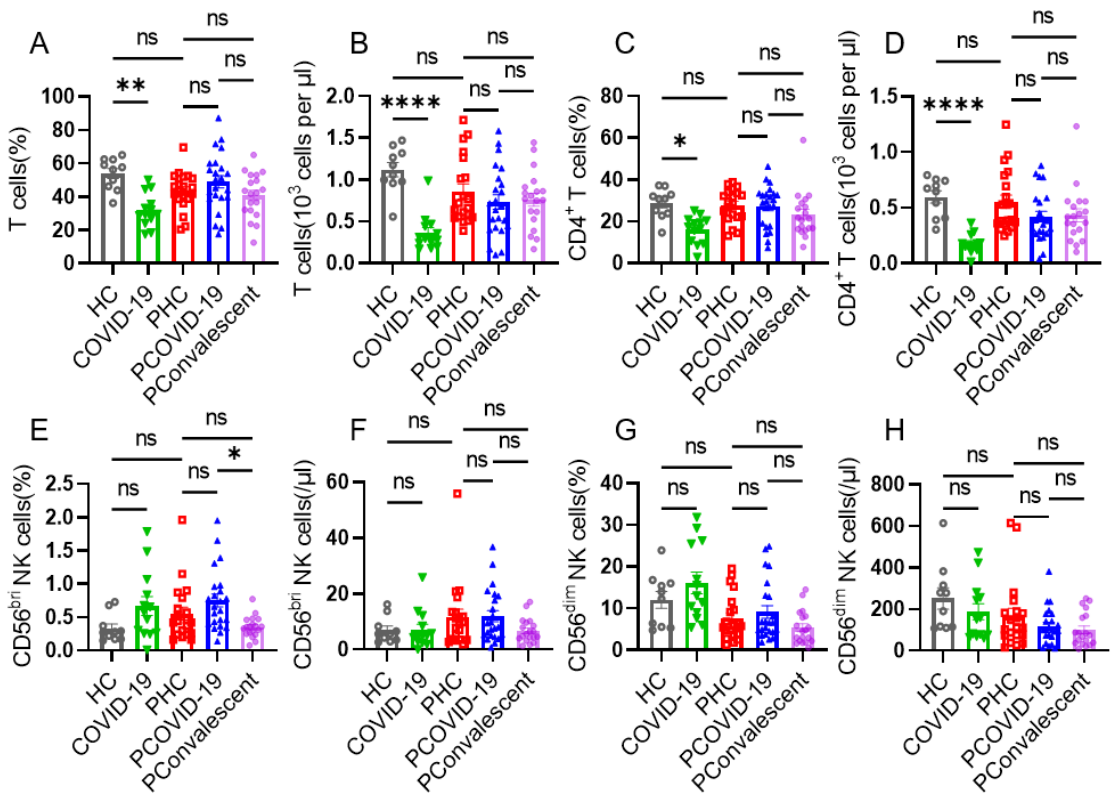

Supplementary Figure 2. The ratio and cellularity of different lymphocyte among the HC group, COVID-19 group, PHC group, PCOVID-19 group and PConvalescent group. The frequency and absolute count of (A-B) T cells, (C-D) CD4$^+$ T cells, (E-F) CD56^{bri} NK cells and (G-H) CD56^{dim} NK among the HC group, COVID-19 group, PHC group, PCOVID-19 group and PConvalescent group. Results are shown as mean ± SEM. One-way ANOVA was used to determine statistical significance. *P <0.05, **P <0.01, ****P <0.0001, ns, not significant.

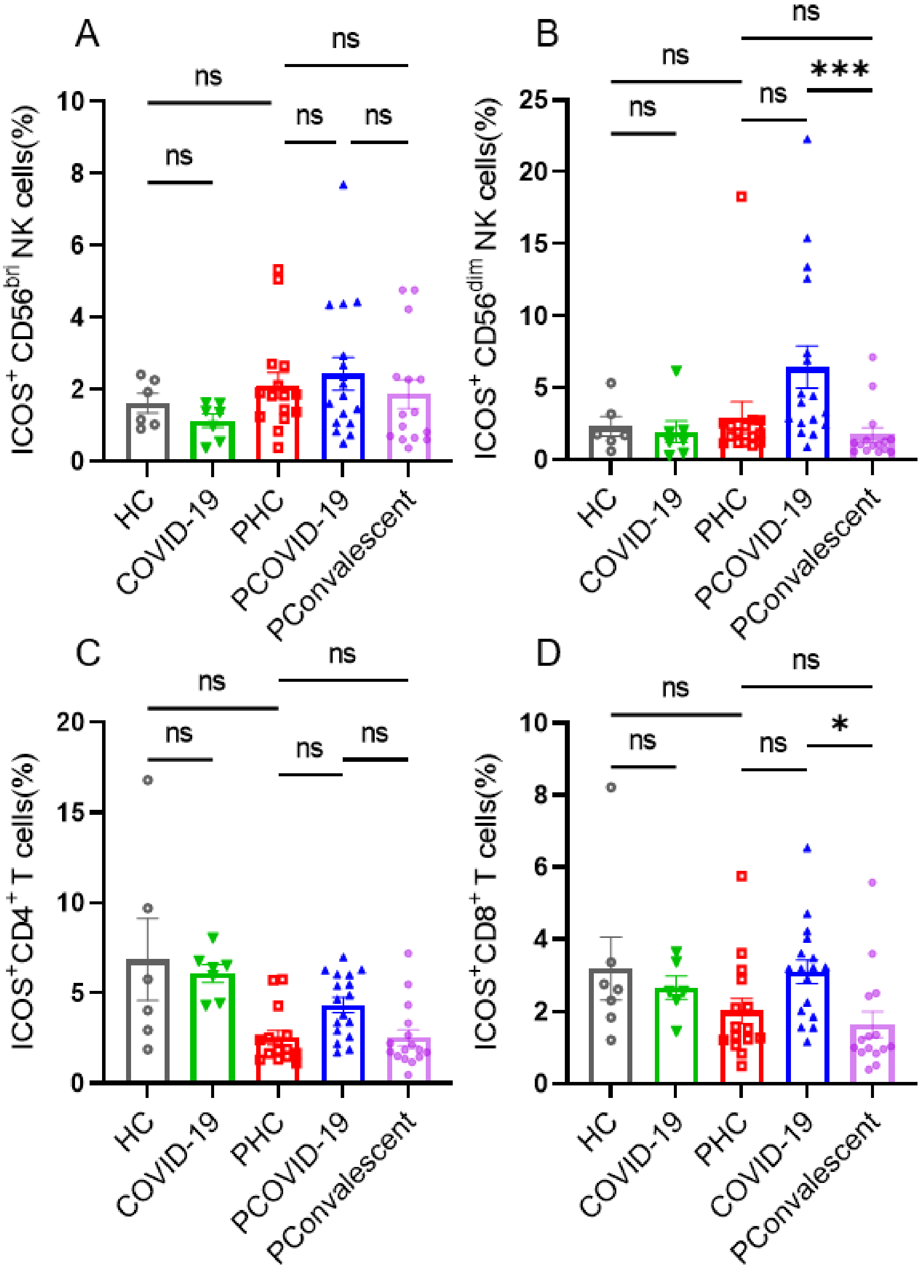

Supplementary Figure 3. ICOS expression in NK cells, $CD4^+$ T and $CD8^+$ T cells among the HC group, COVID-19 group, PHC group, PCOVID-19 group and PConvalescent group. (A-D) The expression of ICOS on (A) $CD56^{bri}$ NK cells, (B) $CD56^{dim}$ NK cells, (C) $CD4^+$ T and (D) $CD8^+$ T cells in HC, COVID-19, PHC, PCOVID-19 and PConvalescent group. Results are shown as mean ± SEM. One-way ANOVA was conducted. * P <0.05; ***P <0.001; ns, not significant.

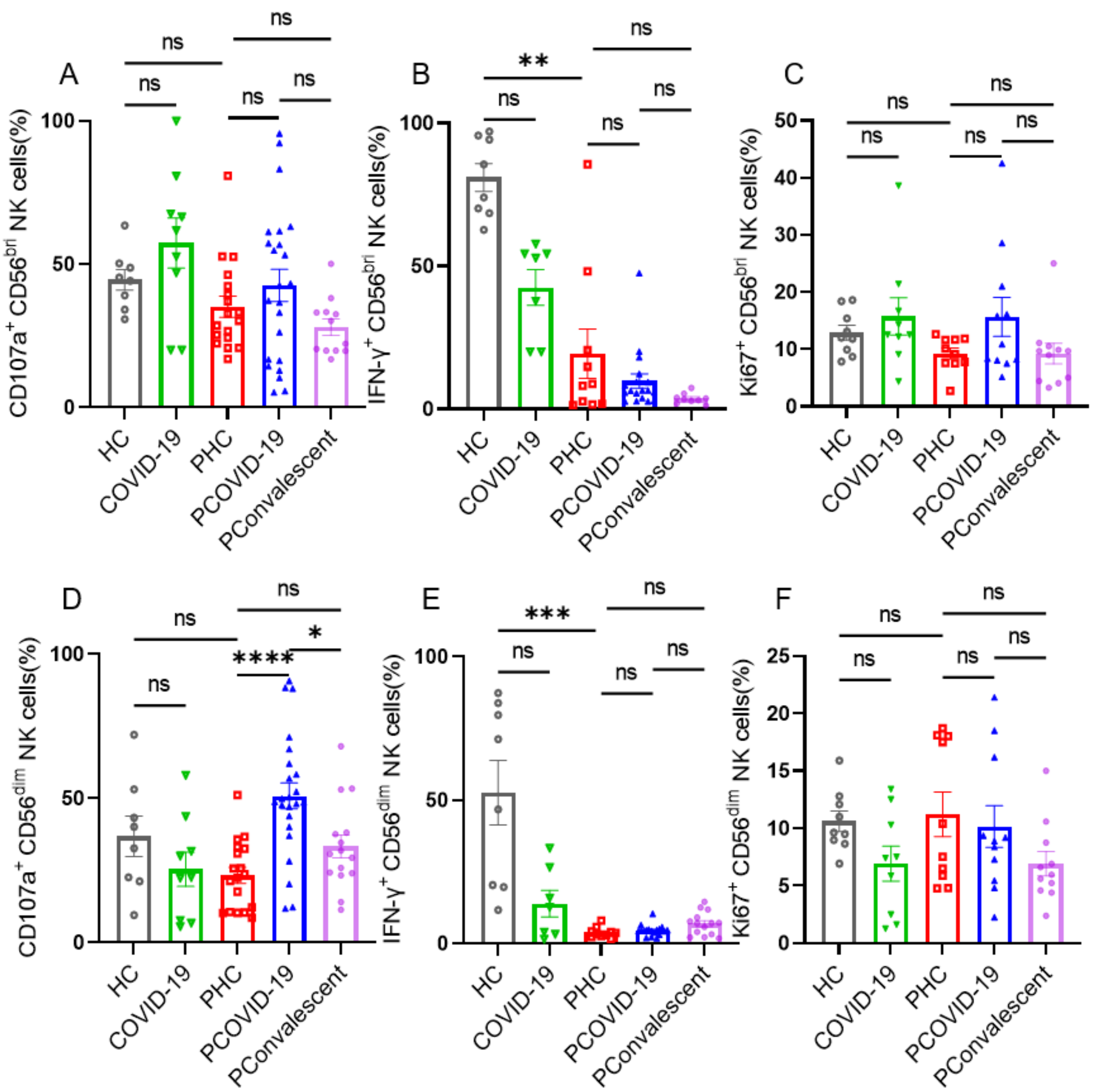

Supplementary Figure 4. The level of cytokine secretion and proliferation ability of NK cells in HC group, COVID-19 group, PHC group, PCOVID-19 group and PConvalescent group. (A-C) The expression of (A) CD107a (B) IFN-γ (C) Ki67 on $CD56^{bri}$ NK cells in HC group, COVID-19 group, PHC group, PCOVID-19 group and PConvalescent group. (D-F) The expression of (D) CD107a (E) IFN-γ (F) Ki67 on $CD56^{dim}$ NK cells in HC group, COVID-19 group, PHC group, PCOVID-19 group and PConvalescent group. Results are shown as mean ± SEM. One-way ANOVA was used to determine statistical significance. *P < 0.05; **P <0.01; ***P <0.001; ****P <0.0001; ns, not significant.

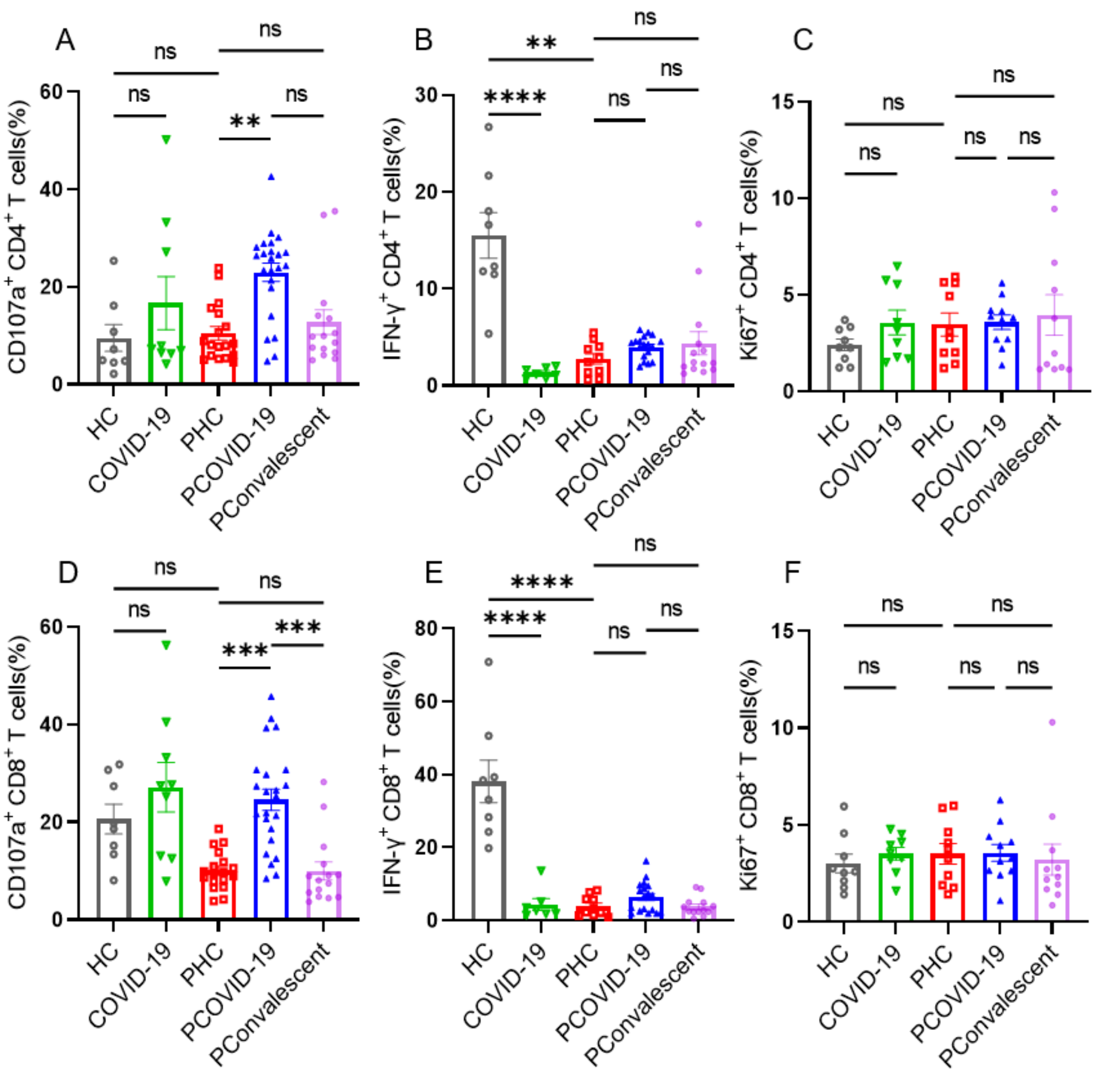

Supplementary Figure 5. The level of cytokine secretion and proliferation ability of T cells in HC group, COVID-19 group, PHC group, PCOVID-19 group and PConvalescent group. (A-C) The expression of (A) CD107a (B) IFN-γ (C) Ki67 on $CD4^+$ T cells in HC group, COVID-19 group, PHC group, PCOVID-19 group and PConvalescent group. (D-F) The expression of (D) CD107a (E) IFN-γ (F) Ki67 on $CD8^+$ T cells in HC group, COVID-19 group, PHC group, PCOVID-19 group and PConvalescent group. Results are shown as mean ± SEM. One-way ANOVA was used to determine statistical significance. ***P* <0.01; ****P* <0.001 *****P* <0.0001; ns, not significant.

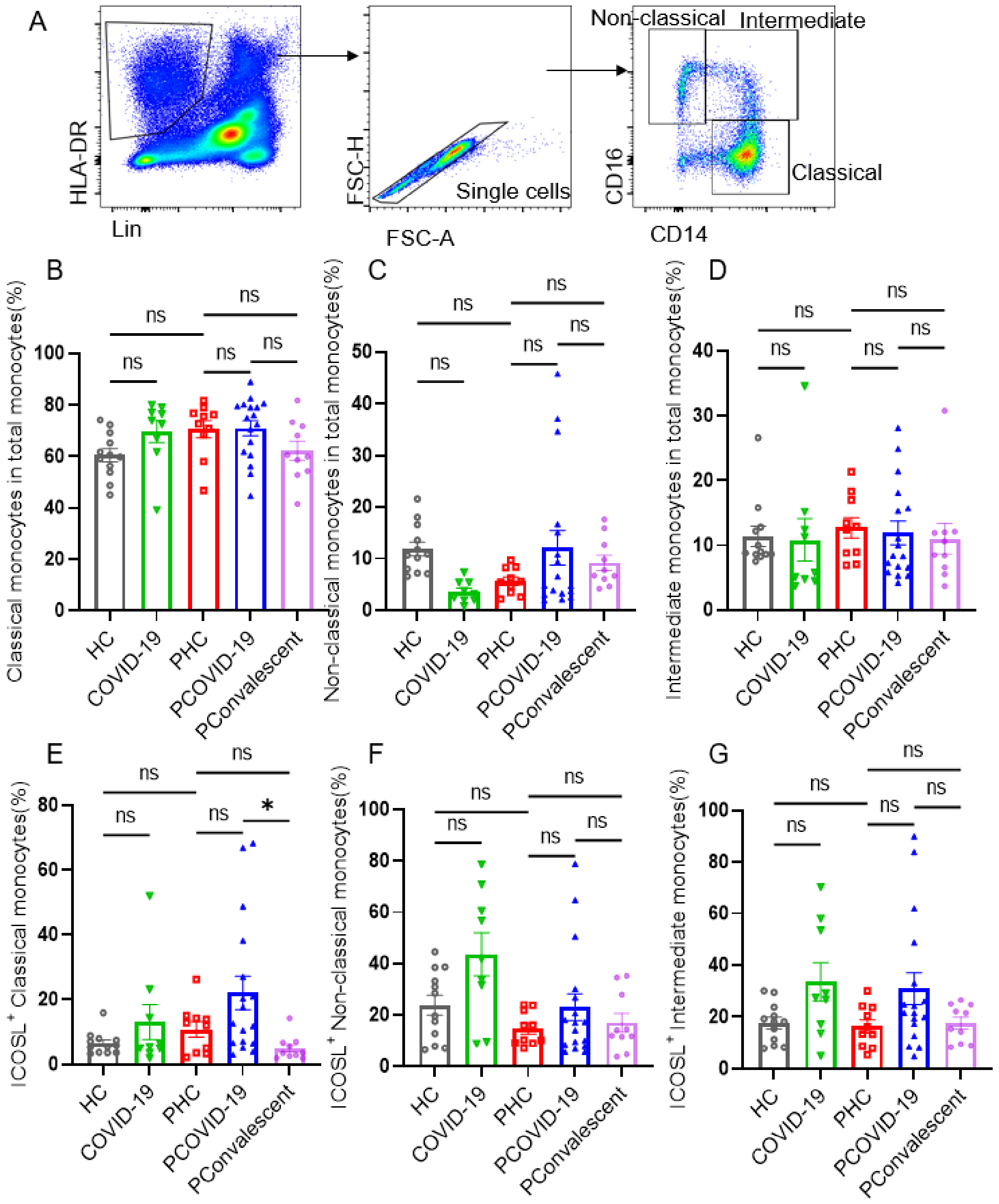

Supplementary Figure 6. Expression of ICOSL among the HC group, COVID-19 group, PHC group, PCOVID-19 group and PConvalescent group. (A) Gating strategy of monocytes, starting with Lin^{-}(CD3, CD19, CD20, CD56, CD66b) HLA-DR^{+}, and then divided it into three groups as $CD14^{+}CD16^{-}$ (classical), $CD14^{+}CD16^{+}$ (intermediate) and $CD14^{-}CD16^{+}$ (non-classical) monocytes. (B-D) The proportion of (A) $CD14^{+}CD16^{-}$ classical monocytes, (C) $CD14^{-}CD16^{+}$ non-classical monocytes and (D) $CD14^{+}CD16^{+}$ intermediate in HC group, COVID-19 group, PHC group, PCOVID-19 group and PConvalescent group. (E-G) The expression of ICOSL in (E) $CD14^{+}CD16^{-}$ classical monocytes, (F) $CD14^{-}CD16^{+}$ non-classical monocytes and (G) $CD14^{+}CD16^{+}$ intermediate in HC group, COVID-19 group, PHC group, PCOVID-19 group and PConvalescent group. Results are shown as mean ± SEM. One-way ANOVA was used to determine statistical significance. $*P < 0.05$; ns, not significant.
